# P-2290. Pulmonary *Nocardiosis* in Renal Transplant Recipients From Pakistan: Risk Factors,Clinical Presentation and Mortality

**DOI:** 10.1093/ofid/ofae631.2443

**Published:** 2025-01-29

**Authors:** Rohama Samar, Sunil Dodani, Jawwad Nazmi

**Affiliations:** Sindh Institute of Urology and Transplantation, Karachi, Sindh, Pakistan; Sindh Institute of Urology and Transplantation, Karachi, Sindh, Pakistan; Sindh Institute of Urology and Transplantation, Karachi, Sindh, Pakistan

## Abstract

**Background:**

Nocardia is an opportunistic infection among renal transplant recipients (RTR) with an incidence of less than 1% but very high mortality. Data from Pakistan is scarce. Our aim is to find the risk factors, clinical and radiographic findings, antimicrobial sensitivity and outcome of Nocardia infection among renal transplant recipients.

**Figure:**
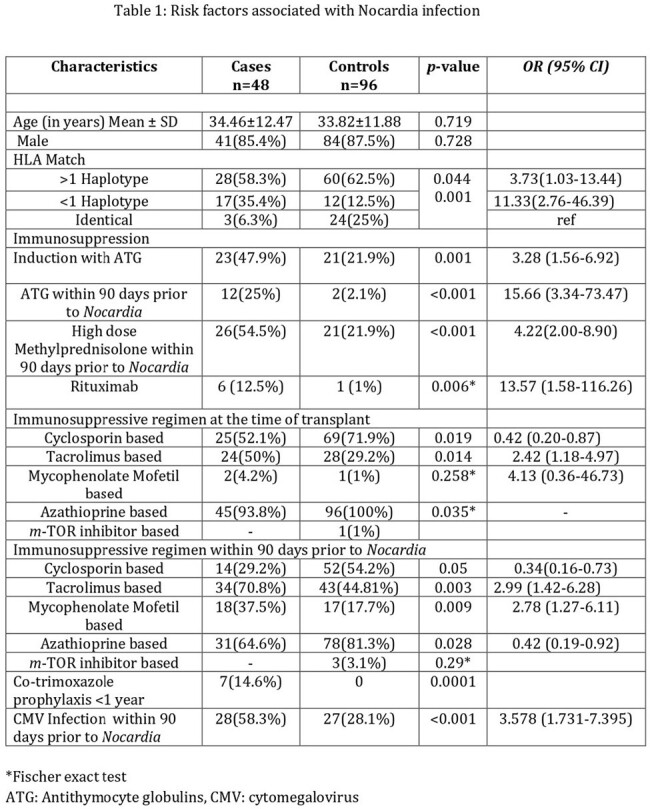

**Methods:**

All adult RTR diagnosed as Nocardia infection between 2013 and 2020 were included. The cases were matched with 1:2 controls based on sex, age (+/- 1 year), and transplant date (+/- 1 year). Risk factors, clinical features, antibiotic sensitivities and outcome were analyzed.

**Figure:**
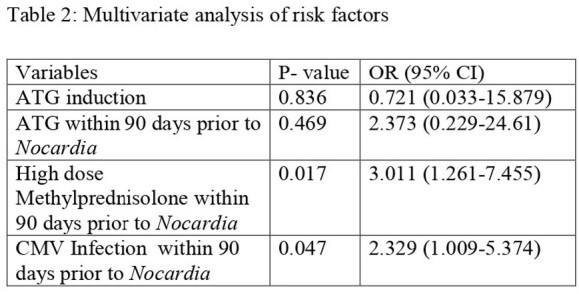

**Results:**

A total of 48 patients developed Nocardia. Around 25% presented with disseminated disease. Median time since transplant was 2.68 years. High dose methylprednisolone and cytomegalovirus (CMV) infection within 90 days were independent risk factors for

Nocardia infection. The mortality rate was 20%. Central nervous system disease (CNS) and CMV infection within 90 days were significantly associated with mortality. The most susceptible drugs were co-trimoxazole and linezolid. Imipenem susceptibility was only 20%.

**Figure:**
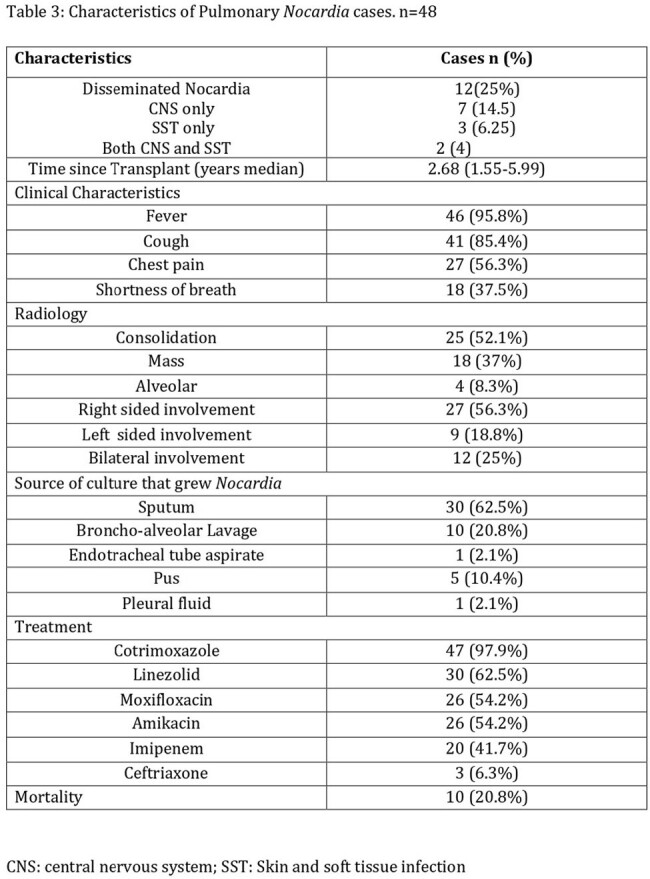

**Conclusion:**

High dose methylprednisolone and CMV infection were independent risk factors for Nocardia infection. CNS disease is associated with mortality. Nocardia species were highly resistant to ceftriaxone and imipenem in our patient population.

**Figure:**
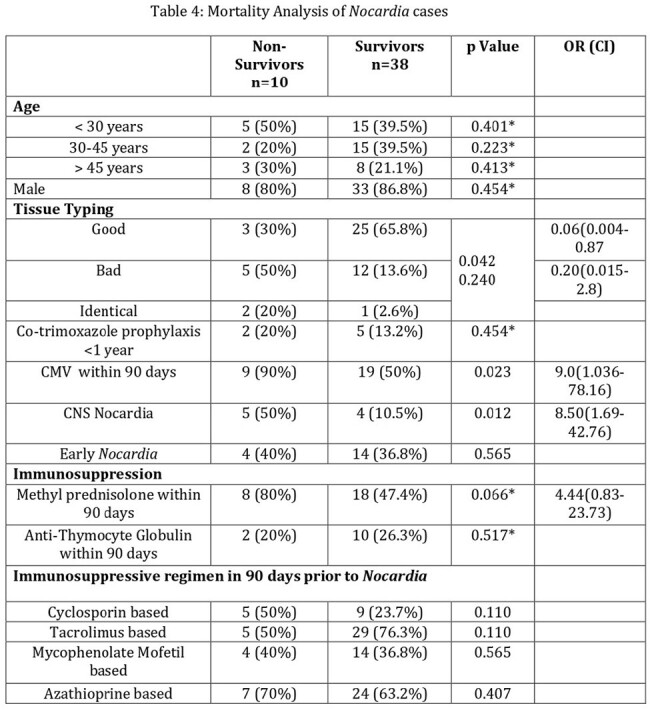

**Disclosures:**

All Authors: No reported disclosures

